# New European Discovery of *Splachnum pensylvanicum* (Bryophyta, Splachnaceae) in Lithuania, with Taxonomic Notes and a Review of Its World Distribution

**DOI:** 10.3390/plants10122823

**Published:** 2021-12-20

**Authors:** Ryszard Ochyra, Ilona Jukonienė, Vítězslav Plášek, Sigita Sprainaitytė

**Affiliations:** 1 Laboratory of Bryology, Władysław Szafer Institute of Botany, Polish Academy of Sciences, Lubicz 46, 31-512 Kraków, Poland; r.ochyra@botany.pl; 2 Nature Research Centre, Institute of Botany, Žaliujų Ežerų Str. 49, 08406 Vilnius, Lithuania; ilona.jukoniene@gamtc.lt; 3 Department of Botany, University of Ostrava, Chittussiho 10, 710 00 Ostrava, Czech Republic and Institute of Biology, University of Opole, 45-040 Opole, Poland; 4 Kamanos Strict Nature Reserve, Pušų Str. 2, Akmenė 2nd Village, 85324 Akmenė District, Lithuania; s.sprainaityte@yahoo.com

**Keywords:** Baltic countries, coprophilous mosses, Euro-American distribution pattern, Kamanos mire, Neotropics, North America

## Abstract

*Splachnum pensylvanicum* (Brid.) Grout *ex* H.A.Crum is recorded for the first time in Lithuania and it is its fourth discovery at a third locality in Europe. It was found for the first time in 2000 in Kamanos mire, the largest peatland complex in the northern part of this East Baltic country. Targeted investigations at this site in 2017 resulted in the discovery of 14 populations and it is apparently the largest and most abundant locality of the species in Europe. *Splachnum pensylvanicum* is briefly described and illustrated along with some taxonomic notes and a detailed description of its habitat requirements. The global geographical distribution of *S*. *pensylvanicum* is reviewed and mapped. It is a Euro-Eastern North American temperate species and deeply penetrates into the Neotropics at montane stations in Venezuela and SE Brazil in South America.

## 1. Introduction

Most species of splachnalean mosses are unique in their ecological predilections, being associated with nitrogen-enriched organic substrates, usually in damp habitats in peatlands. As typical nitrophytes, they are often coprophytes that grow on the droppings, faeces, and dung of large mammals, but many of them thrive also on old bones, pellets and decaying carcasses of animals. Less often they can be found on rotten wood, humus and wooded soil. For some species of *Splachnum* Hedw. evidence of entomophily was provided [1,2,3,4,5,6,7,8,9]. They have many unusual adaptations for the dispersal of spores that allure coprophilous flies, including brightly coloured and expanded hypophyses of the capsules which emanate odours and volatile compounds and eject secretions [10,11,12,13]. Moreover, they commonly produce small and sticky spores which are easily dispersed to fresh droppings by dipteran flies, and where the spores germinate very easily, no matter what concentration of minerals is available in these substrates [14]. In addition, species of *Splachnum* can better tolerate high ionic concentrations in the substrate and consequently they have a competitive advantage over other species of splachnalean mosses [15,16,17].

Despite growing on quite unattractive substrates, morphologically the splachnalean species are handsome and attractive mosses, not only for some flies, but also for bryologists. It is therefore no wonder that one of the earliest, if not the first, printed description and figures of a splachnalean moss was published by J. Petiver as early as in 1695 under the polynomial Latin name *Muscus Norwegicus umbraculo ruberrimo insignitus* and the common name “Bongrace moss” [18]. His description and figure, with the unmistakable umbrella-like hypophysis, leave no doubt that it was a *Splachnum* to which he referred. Furthermore, according to Petiver’s glossary, the asterisk in the margin next to the description “[…] shews it not to have been known before.” This species was later identified as *S. rubrum* Hedw. [19,20] and, together with *S. luteum* Hedw. it is the most beautiful and most conspicuous moss of the northern Holarctic coniferous forests.

However, the most often treated species of *Splachnum* at that time was *S. ampullaceum* Hedw. as it is the most frequent species of the genus in Europe, widespread in the boreal and northern temperate zones [21]. Actually, its earliest name, *Adiantum aureum minus palustre, capitulus erectis coronatis*, was used for a plant collected by W. Sherard near Southampton in England, and this name was published, but without description, in 1690 in the first edition of *Synopsis methodica stirpium britannicarum* [22]. A short description of this moss, but without an icon, was published in 1696 in the second edition of this opus [23]. An expanded description accompanied by the first illustration of *S. ampullaceum* was presented only in 1699 by R. Morison [24].

Knowledge about the mosses of the Splachnaceae up to 1740 was summarised in 1741 by J. J. Dillenius in his famous *Historia muscorum* [25], an opus that actually gave rise to research on bryophytes. He described and illustrated five species which are currently placed in this family, including *Splachnum ampullaceum*, *S. sphaericum* Hedw., *S. pensylvanicum* (Brid.) Grout *ex* H.A.Crum, *S. rubrum* and *Tetraplodon angustatus* (Hedw.) Bruch & Schimp. for which only polynomial names had been given [20].

At the very beginning of the nineteenth century, in his flagship work *Species muscorum frondosorum*, now considered the starting point for the nomenclature of mosses (except *Sphagnum* L.), J. Hedwig described 12 species of *Splachnum* [19], which currently belong to three genera of the Splachnaceae, namely *Tetraplodon* Bruch & Schimp., *Tayloria* Hook. and *Splachnum*. The best measure to draw special attention to species of the Splachnaceae at that time may have been the fact that, with the exception of two, all species of this family known from Europe had been described by the year 1840. 

Currently, 22 species of the Splachnaceae are known to occur in Europe, seven of which belong to the genus *Splachnum*. The rarest species, not only of this genus but also of the whole family, is *S. pensylvanicum*, which has been collected only three times in the East Baltic region [26,27]. Two collections come from Kaliningradskaya Oblast’ (=“Kaliningrad Province”) in Russia [28,29] and one from Latvia [30,31,32,33]. Herein, the species is recorded from Lithuania where it was discovered for the first time in 2000 in Kamanos mire, the largest peatland complex in Akmenė district, in the northern part of the country next to the border with Latvia.

Although *Splachnum pensylvanicum* is firmly established as a member of the moss flora of Europe, it has not been described in detail based on European material. Likewise, in the recently published photographic flora of Europe’s mosses, illustrative material based on specimens from Brazil is presented [34]. To remedy this deficiency, this study provides a description and illustrations of this species fully based on the specimens collected in Lithuania. Additionally, the European and world distribution of *S. pensylvanicum* are reviewed and mapped. 

## 2. Materials and Methods

### 2.1. Herbarium Materials

The herbarium materials have been deposited in BILAS and KRAM. During the course of the present study, the type material of *Tayloria baltica* Warnst. (in B) and the Latvian voucher material located in the Latvian State Forest Research Institute “Silava” in Salaspils were studied. The global distribution of *Splachnum pensylvanicum* is based primarily on herbarium holdings of the species deposited in NY. 

### 2.2. Description of the New Locality

Kamanos is the largest mire complex in northern Lithuania. It is situated at lat. 56°14′40″–56°19>′18″ N and long. 22°34′20″–22°43′45″ E at an elevation of 80–90 m a.s.l. The peatland includes raised peat bogs, fens, transitional mires, Lake Kamanos (area about 6 ha), more than 120 small pools and surrounding wet forests. The bogs cover the largest area—1722 ha. The depth of peat bed reaches 7.2 m (average 3.78 m). Since 1979, Kamanos mire with the surrounding forests (about 4000 ha in total) has been protected as a State Strict Nature Reserve. Since 1993, it has been a Ramsar territory [35,36].

In 1924 the vegetation of nine East Prussian and Lithuanian peatlands, including that of Kamanos mire, was surveyed by two German botanists, Hermann Reimers and Kurt Hueck, within the project of the natural and cultural history of Lithuania and neighbouring areas. The results of this survey were published in 1929 [37]. After a few years, the vegetation of this mire was explored in detail and described by Lithuanian botanists [38,39]. In both monographs, no *Splachnum* species were recorded. Moreover, we have not found any species of *Splachnum* from Kamanos deposited at the Lithuanian herbaria (BILAS and WI) until now.

**Figure 1:**
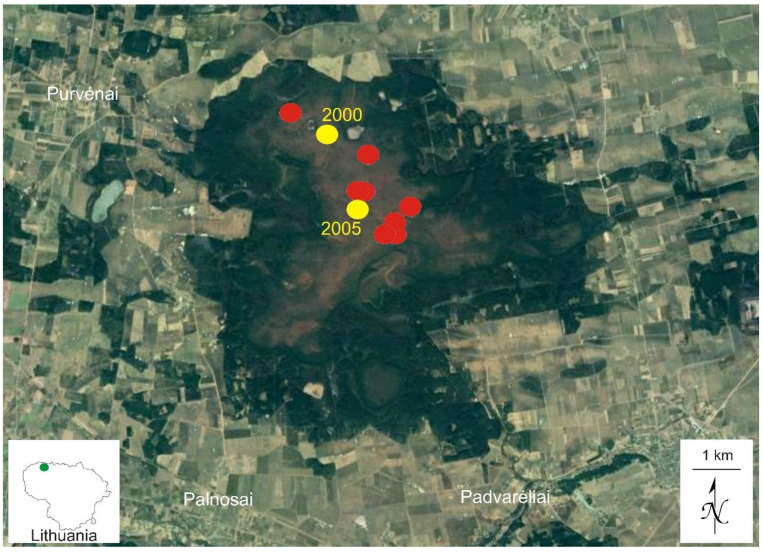
Distribution of *Splachnum pensylvanicum* in Kamanos mire and in Lithuania (inset). The records made in summer–autumn 2017 are marked with red dots.

The first specimen identified as *Splachnum pensylvanicum*, after a revision of unstudied bryophyte collections from Kamanos, was collected in 2000 in the northern part of the mire by I. Jukonienė. In July 2017, after the targeted investigations in the mire, the species was recorded east from the first locality (Figure 1). 

In July–October 2017, fourteen populations (patches) of *Splachnum pensylvanicum* were recorded by S. Sprainaitytė. Most of them were found in the central part of the mire, near the site of the one of the first records of the species (Figure 1). 

## 3. Results and Discussion

### 3.1. Characterisation of Lithuanian Plants

**Figure 2:**
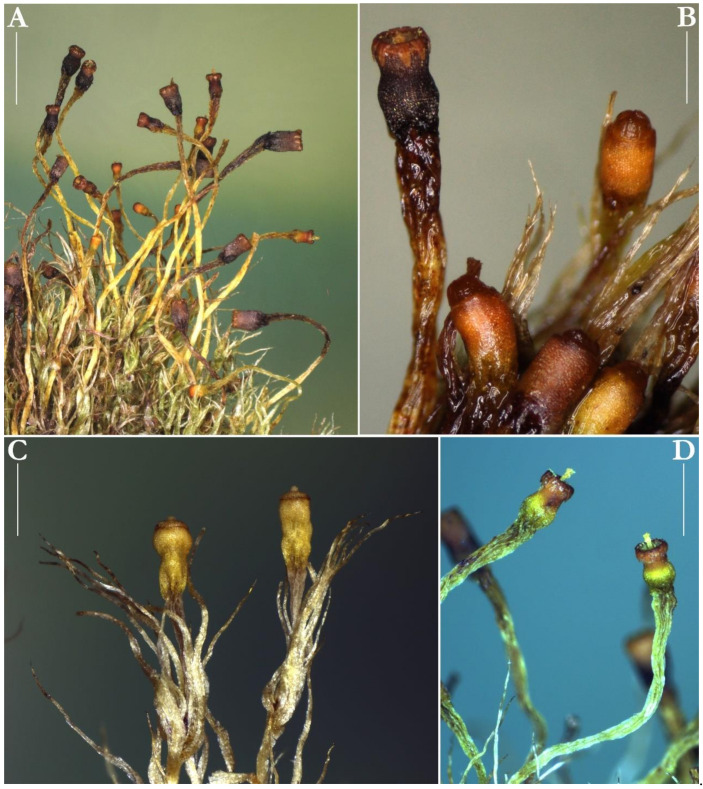
Macro photographs of *Splachnum pensylvanicum* taken from the specimens from Kamanos mire in Lithuania: (**A**) portion of dry tuft showing the plants with numerous mature sporophytes, (**B**) mature deoperculate capsules, (**C**) mature operculate capsules, (**D**) two deoperculate capsules with expanded columellae. All taken from KRAM B-242426 (**A**,**B**,**D**) and KRAM B-242425 (**C**). Scale bars: (**A**)—3 mm, (**B**)—0.5 mm, (**C**,**D**)—1 mm.

**Figure 3:**
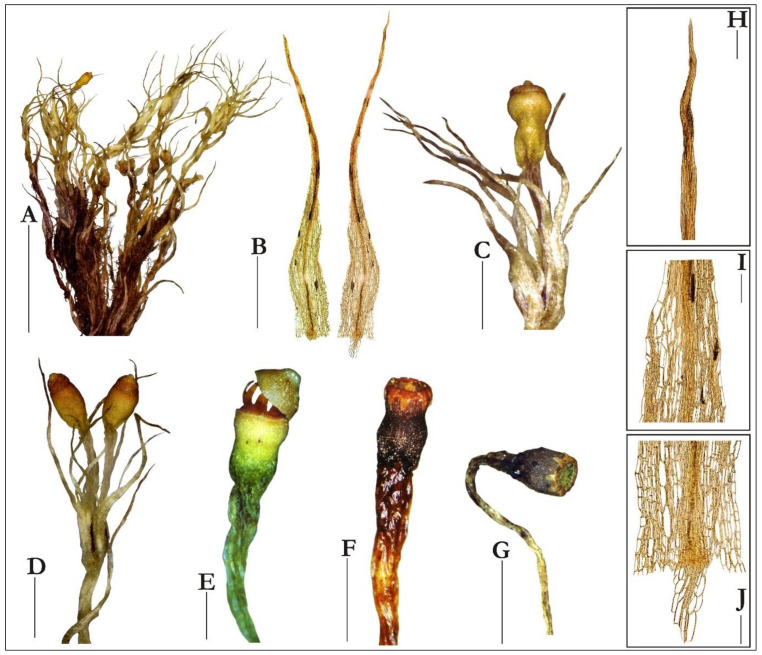
Macro and micro photographs of *Splachnum pensylvanicum* taken from the specimens from Kamanos mire in Lithuania: (**A**) portion of dry tuft showing the plants with numerous mature sporophytes and gametangia, (**B**) upper leaves, (**C**) portion of shoot with mature operculate capsule, (**D**) shoot with two operculate capsules, (**E**–**G**) deoperculate capsules, (**H**) apex of leaf subula, (**I**) upper cells at leaf shoulder, (**J**) basal cells. All taken from KRAM B-242425 (**A**–**D**; **H**–**J**) and KRAM B-242426 (**E**–**G**). Scale bars: (**A**)—2 cm, (**B**)—0.5 mm, (**C**)—1.5 mm, (**D**)—1 mm, (**E**–**F**)—0.5 mm, (**G**)—1.5 mm, (**H**–**J**)—100 µm.

**Figure 4:**
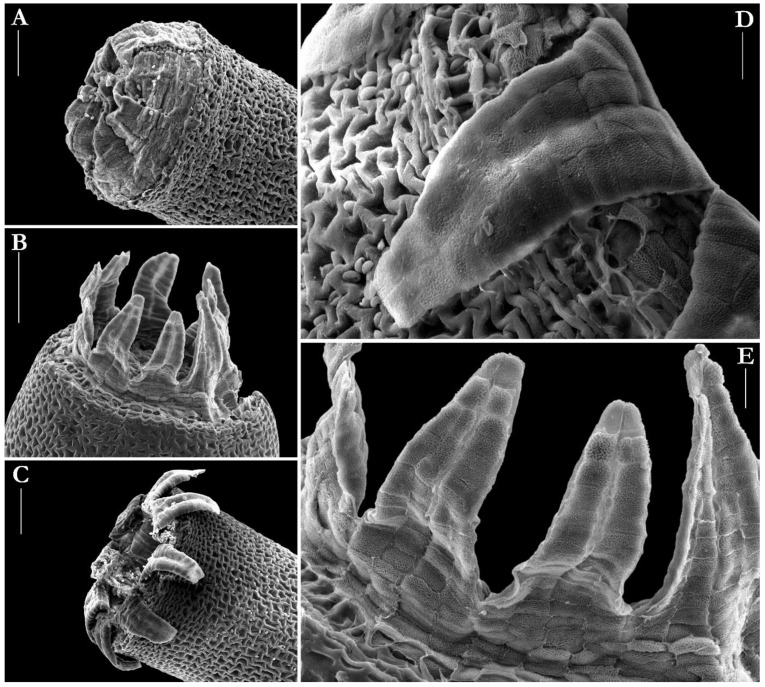
SEM micrographs of the capsule details of *Splachnum pensylvanicum*: (**A**) upper part of the urn showing peristome, spores and upper exothecial cells, (**B**) inflexed peristome teeth, (**C**) reflexed peristome teeth, (**D**) reflexed peristome tooth showing sculpture of its inner surface, (**E**) sculpture of the outer surface of the peristome teeth. All taken from KRAM B-242425. Scale bars: (**A**–**C**)—100 µm, (**D**,**E**)—20 µm.

Careful examination of the voucher specimens of *Splachnum* from Kamanos mire revealed their perfect correspondence with the European and North and South American collections of *Splachnum pensylvanicum*. It is evidenced by the following description and the illustrations of the essential diagnostic characters (Figure 2, Figure 3 and Figure 4).

The Lithuanian material of *S. pensylvanicum* consists of medium-sized, soft and slender, loosely to densely tufted plants that are lustrous, light to yellow-green or vividly green and densely matted with a tomentum of dull red-brown to purple, smooth to somewhat warty and branched rhizoids below. The stems are thin, gracile and flaccid, erect, 1–6 cm tall, simple or branched, rounded to angled in the transverse section, with a distinct central strand, 3–4-stratose medulla of enlarged, hyaline, thin-walled cells and a poorly developed or lacking cortex, usually with distinct false leaf traces. The axillary hairs are 2–3-celled, with 1–2 subquadrate, red basal cells and an oblong, swollen, hyaline upper cell. The leaves are distant, but usually more crowded toward the stem apices, loosely erect to spreading when wet, flexuose-contorted to somewhat twisted on drying, 2.0–3.5 mm long, 0.4–0.6 mm wide, often reaching to the capsules and sometimes exceeding them, long-lanceolate and very slenderly long-acuminate above, and ovate to obovate and rather abruptly short to long-acuminate in the lower part of the stem. The leaf margins are plane, almost entire in the upper leaves and sparsely toothed with scattered, large and many-celled teeth in the lower leaves. The costa is single, stout and vanishes in the acumen near the apex or more often almost fills the subula, in the transverse section with enlarged, thin-walled adaxial and abaxial epidermal cells and well-developed hydroids, but without stereids and guide cells. The laminal cells are thin-walled, lax and smooth, rectangular to elongate-hexagonal above, 20–60 µm long, 10–16 µm wide, becoming short-rectangular and bulging in the basal part. 

The plants are dioicous or autoicous. The perigonia are cupulate and large and the perichaetia are terminal on the stems or branches with leaves fewer and smaller. The setae are weak and slender, 4–10 mm long, smooth, curved or flexuose, soft and almost hyaline or pale and greenish-white to yellowish. The capsules are exserted or sometimes barely emergent, 1.2–2.5 mm long, obloid-cylindrical and slightly contracted below the mouth, suddenly narrowed to the hypophysis, orange to orange-brown, becoming dark red with age, with the mouth often forming a ridge when dry.

The hypophysis is variable in size, 1–2 mm long, as long as the urn or somewhat shorter or longer, sometimes up to twice as long, as wide or slightly wider than the urn, wrinkled, greenish to purplish. The exothecial cells are thick-walled and more or less collenchymatous, somewhat smaller, oblate, oblate-hexagonal to irregularly triangular in many tiers below the orifice, becoming irregularly hexagonal, elongate to rounded or irregular in shape to the hypophysis, whose cells are long and thinner-walled. The stomata are few, situated at the junction of the urn and the hypophysis and are phaneroporous and consist of two guard cells. The operculum is low-convex or hemispheric and bluntly apiculate. The columella projects above the mouth when dry. The peristome is double but the endostome is fused to the exostome. It consists of 16 teeth united into eight pairs inserted within the mouth. They are reflexed on drying and inflexed on wetting, triangular and blunt at the apex, 320–450 µm long, light- to orange-brown, densely papillose on the outer surface and minutely roughened on the inner surface. The spores are ellipsoid, slightly longer than broad, small, 7–9(–11) µm in diameter, light-green, smooth. The calyptra is short, to 0.5 mm, conic-mitrate, not split or constricted below and barely covers the operculum. 

### 3.2. Habitat and Substrate Preferences

In most cases, *Splachnum pensylvanicum* was found in open, treeless parts of the Kamanos mire. The first specimens were collected in quite dry bog habitats overgrown by *Calluna vulgaris* (L.) Hull. Subsequent records showed that the species also occurs in wetter areas. In wet places, it occupied low hummocks or the lower part of higher hummocks. It was found growing in lawns of the traditionally conceived *Sphagnum magellanicum* Brid., whereas the hummocks were built mainly by *S. magellanicum*, and less often by *S. rubellum* Wilson. The populations of *Splachnum pensylvanicum* formed mostly pure stands, only sometimes intermixed with *S. ampullaceum* Hedw. These two species of *Splachnum* were mostly accompanied by the liverworts *Kurzia pauciflora* (Dicks.) Grolle and *Mylia anomala* (Hook.) S.Gray, and by *Odontoschisma fluitans* (Nees) L.Söderstr. & Váňa in wetter places. 

### 3.3. Taxonomic Remarks

*Splachnum pensylvanicum* is distinguished from the other European congeners by sporophyte and gametophyte traits. Its sporophyte is of a relatively diminished size, with a very short seta, making the capsules shortly exserted or only barely emergent and with a small, scarcely inflated hypophysis. Gametophytically, *S. pensylvanicum* is very similar to *S. ampullaceum* and the two species share long-lanceolate and slenderly acuminate leaves with spinose-dentate, multicellular teeth on the upper leaf margins. This feature enables them to be distinguished when sporophytes are missing. However, while in *S. ampullaceum* such multicellular leaf serration is an outstanding and consistent feature of the leaf margins, it is only sporadically encountered in *S. pensylvanicum*, especially in the upper leaves. This is a particularly prominent feature of the European populations of *S. pensylvanicum*, which have consistent entire margins in the upper leaves. Specimens with sporophytes do not pose any problems with identification, because *S. ampullaceum* differs at a glance in that it has a large, light purple or lilac, inflated and pomiform hypophysis that is always considerably wider than the urn.

In the shape of the leaves and their serration at the margins in the upper part, *Splachnum pensylvanicum* resembles *Tetraplodon angustatus*, where the marginal teeth are unicellular and rather blunt. Additionally, the two species share a similar hypophysis that is elongate and wrinkled and only somewhat inflated and slightly wider than the urn. Based on these characteristics, early researchers have very often combined *S. pensylvanicum* with the genus *Tetraplodon* Bruch & Schimp., including *T. caulescens* (Turton) Lindb. and *T. australis* Sull. & Lesq. *ex* Sull., both now considered conspecific with *S. pensylvanicum* [28,40,41]. In fact, the European populations of this species were first described as *Tetraplodon balticus* Warnst. [42] and *S. pensylvanicum* itself was also transferred to this genus as *Tetraplodon pensylvanicus* (Brid.) Sayre [43].

Despite these striking morphological similarities in the shape of leaves and capsules, this species is much more closely related to the genus *Splachnum* when some structural affinities and the origin of the peristome teeth are considered. The peristome in *Splachnum* is double, but the endostome is fully fused to the 16 exostome teeth, which are united into eight pairs, and as a result the teeth, in the transverse section, are chambered. Each tooth-pair has four columns of cell remnants on its outer surface and four or more columns on its inner surface. In contrast, the peristome in *Tetraplodon* is single and each tooth has two columns of plates on the inner surface and is solid in the cross-section. The chambered structure of the peristome teeth prompted Crum [44] to transfer *Tetraplodon pensylvanicus* to *Splachnum*, and this taxonomic conclusion is well supported by the results of molecular studies [45].

### 3.4. Biological and Ecological Peculiarities

The Lithuanian plants of *Splachnum pensylvanicum* exhibit some distinctive biological and ecological features in comparison with North American populations of the species. In North America, the species produces the mature capsules in the winter–spring season [46]. In Lithuania, the individuals collected at the beginning of July in different years (2000, 2005 and 2017) possessed immature sporophytes with all capsules having well developed lids. However, the specimens collected in August and September were in most cases with fallen opercula, but the spores were still present inside the capsules. Finally, in the specimens collected in October, the spores were already dispersed and the capsules remained empty. So, it seems likely that in Lithuania, the summer–autumn season is the most favourable for maturing the capsules.

In North America, the species usually grows on the dung of herbivores. In Kamanos mire, for the most part it was found on the droppings of common cranes (*Grus grus* Linnaeus) (ten records) and it was only recorded twice on the dung of herbivorous animals. It was recorded growing on open degraded peat, and finally it was also found on a decaying stump of pine. The substrate preferences can explain the local distribution of *Splachnum pensylvanicum*. The central part of Kamanos mire, where most localities of the species are concentrated, is a preferred site for gathering common cranes. 

Despite the fact that *Splachnum pensylvanicum* was not recorded during the botanical studies in the 1920s, it cannot be said that it did not exist in the mire at that time and may have simply been overlooked. The history of the records has revealed that the species has been surviving in Kamanos for at least 17 years and is widespread at present. The status of the strict nature reserve of the mire is conducive for the distribution of the species as it provides suitable habitats for birds and wild animals.

### 3.5. Global Distribution of Splachnum Pensylvanicum

**Figure 5:**
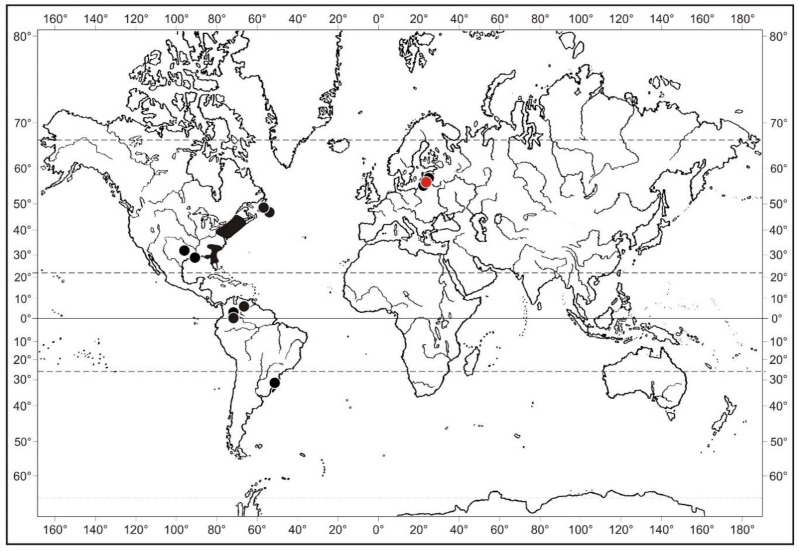
Global distribution map of *Splachnum pensylvanicum*.

**Figure 6:**
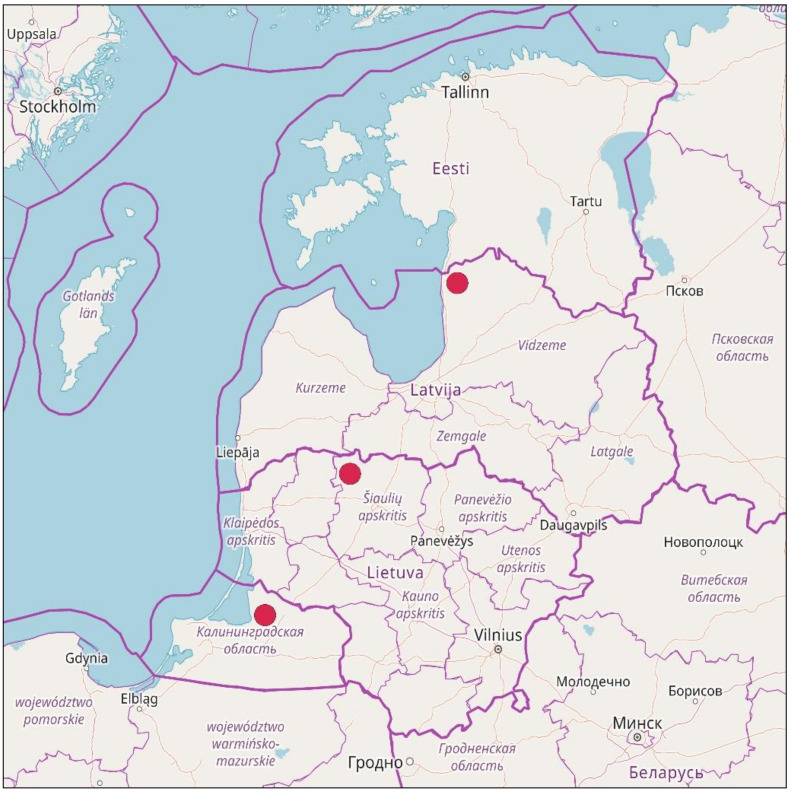
Distribution map of *Splachnum pensylvanicum* in the East Baltic countries.

*Splachnum pensylvanicum* is a disjunct Euro-Eastern North American species deeply penetrating into the Neotropics where it occasionally occurs at montane elevations in Venezuela and SE Brazil (Figure 5). In Europe, the species is exceedingly rare and is currently known to occur at three localities in the East Baltic countries, including Latvia, Lithuania and the Russian Federation (Figure 6). A small population was found for the first time in 1911 by Hugo Gross in the peatland “Große Moosbruch” in the then East Prussia [47] which is a very important mire for the reconstruction of the Holocene history of the vegetation in this region [48]. Nowadays, it is situated in the Kaliningradskaya Oblast’ in the Russian Federation in an area called Bolshoe Mokhovoe Boloto (=Great Moorland). The plants collected by H. Gross were described by C. Warnstorf as a new species, *Tetraplodon balticus* [42], which was subsequently considered identical to *S. pensylvanicum* [28]. The species was rediscovered at the same mire on 11 July 1930 by Karl and Fritz Koppe [29], but it seems that it has not been rediscovered at this locality since then [49]. It has been 63 years since then, until *Splachnum pensylvanicum* was found again in this region, namely in the northwest of Latvia [30], and seven years later also in Lithuania. The last discovery is detailed in the present account.

In the bryological literature, one can find puzzling information about further collections of *Sphagnum pensylvanicum* in Europe. In the moss flora of eastern North America, at the end of the commentary on this species, there is the information that “Frisvoll [...] has written to us [Crum and Anderson] that several European collections have been made from “pellets containing hair and bone of small rodents, etc.”” [50]. Unfortunately, the author of this information has not published any detailed data on this subject anywhere, especially where these “several European collections” came from.

The IUCN Red List of Threatened Species, Version 2019-2 contains information about the occurrence of *Splachnum pensylvanicum* in the Ślęża Massif [*germ.* Zobten] in Poland’s Lower Silesia [50]. Unfortunately, this is false information based on a misunderstanding of the German text in the paper of H. Reimers and K. Hueck published in 1929. In the footnote on p. 472 these authors clearly stated that this locality applied to *Tetraplodon angustatus* [37]. They believed that the new species *T. balticus*, described from East Prussia, did not differ from *T. angustatus*: “Auf die Gross’schen Fundstücke hat Warnstorf [...] eine neue Art, *Tetraplodon balticus*, gegründet, die aber von *T. angustatus* nicht verschieden sein dürfte [“Warnstorf [...] recognised a new species, *Tetraplodon balticus*, on the basis of Gross’s finds, which, however, should not be different from *T. angustatus*”] [37].

Because there is insufficient appropriate data on the abundance, population size and trends of *Splachnum pensylvanicum* to allow a direct or indirect assessment of its risk of extinction in Europe, this species is classified in the Data Deficient (DD) category on the IUCN Red List of Threatened Species [51,52]. However, the status of endangered species has been granted to it in Latvia [27]. The distribution pattern of *S. pensylvanicum* and its abundance at the only Lithuanian station show that the species is not endangered in the country at present, although the population trends need to be observed in the long term.

*Splachnum pensylvanicum* has its maximum occurrence in eastern North America. In contrast to other species of the Splachnaceae, which have a predominantly arctic-boreal distribution [53], *S. pensylvanicum* distinctly has a much more southerly distribution in eastern North America (Figure 5). It grows on old dung of herbivores, especially cows, less often horses and mules, in mires and bogs. It has a more or less continuous range from southern Newfoundland, New Brunswick, Nova Scotia and Québec in Canada [45], through the northern states in the USA (Maine, Massachusetts and New York) to Georgia and Florida in the South with extensions to Louisiana and Texas in the West. It occurs mainly in the Coastal Plain and occasionally penetrates to the uplands in the Southern Appalachians.

As is the case with many Holarctic moss species, *Splachnum pensylvanicum* deeply penetrates into the tropics in South America. It has been recorded several times on tepuis in Venezuela at an elevation of 1690–2300 m [54], and once it was discovered in fen at 1730 m in the Urubici region in Santa Catarina province in SE Brazil [44].

The occurrence of *Splachnum pensylvanicum* in Europe may seem strange at first sight, but this type of range is represented by a number of moss and liverwort species [55], which indicates that it has a deeper historical background. The phenomenon of the occurrence of species from the Southern Appalachian Mountains was already noted in 1941 by A. J. Sharp [56]), and subsequent discoveries confirmed that such a geographical range is shown by a small group of noteworthy species. Among mosses, the most typical examples are *Anacamptodon splachnoides* (Brid.) Brid., *Brachythecium novae-angliae* (Sull. & Lesq.) A.Jaeger, *Clasmatodon parvulus* (Hampe) Sull., *Dichelyma capillaceum* (Dicks.) Myrin, *Ephemerum spinulosum* Schimp., *Haplocladium virginianum* (Brid.) Broth., *Pelekium minutulum* (Hedw.) Touw, *Sphagnum angermanicum* Melin and *Alleniella complanata* (Hedw.) S.Olsson, Enroth & D.Quandt. Like *S. pensylvanicum*, the latter species also penetrates into the tropics in East and South Africa [57,58].

**Selected specimens examined.** EUROPE. **Russia**. ***Kaliningrad Oblast***: Slavsky district, Bolshoe Mokhovoe Boloto (*germ.* Große Moosbruch), south-east of the village of Gromovo (*germ.* Lauknen) east of the Curonian Lagoon, alt. *ca* 5 m, 54°57′ N, 21°26′ E, on very wet slopes on a footpath leading to the moor, in a small lawn, 24 Aug 1911, *Gross s.n.* (B Bryo-63333; barcode: B 30 0063333, type of *Tetraplodon balticus*). **Lithuania**. ***Akmenė district***: Kamanos Strict Nature Reserve, Kamanos mire: Didžioji klampynė, alt. 81 m, 56°18′39.68″ N, 22°38′42.06″ E, *Sphagnetum magellanici* community, on hummock with *Aulacomnium palustre* and *Sphagnum rubellum*, 22 August 2017, *Sprainaitytė s.n.* (BILAS B-19593, KRAM B-242425); same locality, on the trail of animals, 8 July 2001, *Jukonienė s.n.* (BILAS B-13979, KRAM B-242426), same locality, Didžioji klampynė, alt. 81 m, 56°17′42.71″ N, 22°38′24.33″ E, in Calluna vulgaris dominated community, on herbivore dung, 3 July 2005, Jukonienė s.n. (BILAS B-16106, KRAM B-230536) **Latvia**. ***Vidzeme region***, Limbaži district, Pāle parish: North Vidzeme Biosphere Reserve, Niedrāje–Pilkas Swamp Trail, north-west of Pāle city, alt. 46 m, 57°44′25.75″ N, 24°38′23.93″ E, moss in the swamp by the trail, 16 July 1993, *Bambe 1908* (Hb. Latvian State Forest Research Institute “Silava”, Salaspils).

NORTH AMERICA. **Canada**. ***Newfoundland***: Avalon Peninsula, Hall’s Gully, 5.5 km south on Hall’s Gully Road, alt. ±100 m, 47°21′56″ N, 53°26′59″ W, 8 September 2007, *Buck 52,409* (NY 00944289). ***Nova Scotia***: Cape Breton, Isle Madame, Arichat, 20 July 1882, *Allen s.n.* (NY 00,649,894 & 00653056). **U.S.A. *Maine*:** Washington Co., Town of Beals, Great Wass Island, 27 June 2017, *Buck 64,654* (NY 03075619). ***Massachusetts***: Worcester Co., Brookfield, 14 July 1911, *Dobbin s.n.* (NY 00653110). ***New York***: Essex Co., Town of Minerva, Moxham Pond 2.5 km SW of Olmstedville, 19 September 1993, *Miller 10,523* (NY 1596886). ***New Jersey***: Mercer Co., Quaker Bridge, May 1831, *Greene s.n.* (NY 00649893); Nonmouth Co., Ocean Grove, July 1885, *Underwood s.n.* (NY 00653102); Ocean Co., Cedar Swamps, Nov 1868, *Austin s.n.* (NY 00653104); southern part of the state, Austin, *Musci Appalachiani* No. 177 (1870) (NY 00653099). ***Tennessee***: Johnson Co., Shady Valley, alt. *ca* 854 m, 22 October 1933, *Sharp s.n.* (NY 00653109). ***North Carolina***: New Hanover Co., Wilmington, margin of Gall bays, without collector and date (NY 01475633); without specific locality and date, *Searing s.n.* (NY 00653098). ***South Carolina***: without locality and date, *Rugel s.n.* (NY-Mitten 00653112). ***Louisiana***: New Orleans, Drummond, *Musci Americani* No. 27 (1841) (as *Splachnum setaceum*?) (NY-Mitten 00653111). ***Georgia***: Clinch Co., Fargo, Okefenokee Swamp Refuge, 8 February 1941, *A. J. G. Anderson & L. E. Anderson s.n.* (Grout, *North American Musci Perfecti* No. 411) (NY 00653095); Colquitt Co., 2 mi. N of Moultrie, 24 September 1902, *Harper 1668a* (NY 00653075). ***Texas***: Houston Co., 2 mi. S of Grapeland, 4 March 1950, *Whitehouse 22,845* (NY 00653094). ***Florida***: Leon Co., 5 mi. W of Tallahassee, 15 February 1938, *Schornherst 75* (NY 00653070); Franklin Co., 1.5 mi. E of East Point on Rd. 319, 6 March 1961, *Mitchell 1092* (NY 00653068); Hillsborough Co., Tampa, October 1877, *Garber 324* (NY 00653089); Seminole Co., Sanford, May 1903, *Rapp s.n.* (J. M. Holzinger, *Musci Acrocarpi Boreali-Americani* No. 68) (NY 00653088 & 00653089); Marion Co., Ocala, 17 April 1948, *Schornherst 2451* (NY 01475627); Highlands Co., Avon Park, 31 October 1954, *Brass 25,260* (NY 01475621) *& 25,261* (NY 01475622); Leon Co., Midway Cut-off, 15 February 1939, *Schornherst 75* (NY 01475631). ***New Jersey*–*Florida***: swamps near the sea-coast (without specific locality), Sullivant & Lesquereux, *Musci Boreali-Americani* No. 151 (1856 [1857]) (NY 00653108, syntype of *Tetraplodon australis* Sull. & Lesq.). 

SOUTH AMERICA. **Venezuela**. ***Amazonas***: Dept. Río Negro, Planicie de Zuloaga, Rio Titirico, alt. 2300 m, 10–15 October 1970, *Steyermark 103,922* (NY 01475639); 3.5 km W of Pico Zuloaga, alt. 2000 m, 0°53′ N, 65°56′ E, 13–15 April 1984, *Thomas 3135 & T. Plowman* (NY 01475638); Cerro de la Neblina, 2.8 km NE of Pico Phelps, alt. 2085–2100 m, 0°49′40″ S, 65°59′ W, 29 January 1985, *Buck 12,680* (NY 01475636); Cerro de la Neblina, north-east end of north-west plateau, 12.5 km NNW of Pico Phelps, alt. 1690 m, 0°54′30″ S, 66°02′30″ W, 12–13 February 1985, *Buck 12929A* (NY 01475637).

## 4. Concluding Remarks

*Splachnum pensylvanicum* is a remarkable addition to the moss flora of Europe and Lithuania. The discovery of the fairly abundant populations of this species means that its previous finds in the Kaliningrad Oblast’ and Latvia were not accidental dispersions by man from North America. The species is firmly established in this part of Europe and further findings in this region are possible, not only in the countries in which it is already known, but also in the coterminous areas of Estonia, northern Poland and Belarus. In these countries, following intensive field investigations in recent decades, the local bryophyte floras have been significantly enriched with numerous new country records. 

The best example is Lithuania, which has relatively poor moss flora because of the natural conditions prevailing in this small country, especially the lack of natural rock outcrops. In 2003, some 335 species have been recorded in Lithuania [59], but since then no fewer than 20 species have been added, including the present record of *Splachnum pensylvanicum* [60,61,62,63,64,65,66,67].

## Data Availability

All authors agree with MDPI Research Data Policies.
